# Underwater Acoustic Target Tracking: A Review

**DOI:** 10.3390/s18010112

**Published:** 2018-01-02

**Authors:** Junhai Luo, Ying Han, Liying Fan

**Affiliations:** 1 School of Electronic Engineering, University of Electronic Science and Technology of China, Chengdu 611731, China; hanying@std.uestc.edu.cn (Y.H.); liying_fan@std.uestc.edu.cn (L.F.); 2 Department of Electrical Engineering and Computer Science, The University of Tennessee Knoxville, Knoxville, TN 37919, USA

**Keywords:** underwater target tracking, instrument-assisted method, mode-based method, tracking optimization method, survey

## Abstract

Advances in acoustic technology and instrumentation now make it possible to explore marine resources. As a significant component of ocean exploration, underwater acoustic target tracking has aroused wide attention both in military and civil fields. Due to the complexity of the marine environment, numerous techniques have been proposed to obtain better tracking performance. In this paper, we survey over 100 papers ranging from innovative papers to the state-of-the-art in this field to present underwater tracking technologies. Not only the related knowledge of acoustic tracking instrument and tracking progress is clarified in detail, but also a novel taxonomy method is proposed. In this paper, algorithms for underwater acoustic target tracking are classified based on the methods used as: (1) instrument-assisted methods; (2) mode-based methods; (3) tracking optimization methods. These algorithms are compared and analyzed in the aspect of dimensions, numbers, and maneuvering of the tracking target, which is different from other survey papers. Meanwhile, challenges, countermeasures, and lessons learned are illustrated in this paper.

## 1. Introduction

With the exhaustion of land resources, humans have started shifting their focus to the exploitation and utilization of marine resources for the sake of achieving sustainable development [1]. As a significant component of ocean exploration, underwater target tracking has been a hot area of research in recent years. Tracking is a sophisticated process of estimating the state (i.e., position, velocity, and acceleration) of single or multiple moving targets quickly and as close to the true state as possible by using the available measurements collected from various sensors. It is critical in the war environment for two principal reasons. One is to prevent ourselves from being attacked, and the second is to destroy the enemy. To some extent, the accuracy of acquired information can determine the success or failure of a war. In civil systems, the salvage and rescue of underwater targets and marine life tracking cannot be realized without the techniques of underwater target tracking. Therefore, it is necessary to provide an overview of the state-of-the-art algorithms in underwater target tracking.

A considerable amount of literature has investigated well the problem of target tracking in the terrestrial environment. Among them, the systems based on varieties of sensors can be adopted to detect and track the interested target [2]. In [3], the acoustic sensors are utilized to detect and track targets by judging whether the strength of the received acoustic signal exceeds a predefined threshold. Since vibrations can be used to distinguish targets with different speeds and weights, the system making use of the properties of seismic and passive infrared sensors for detection and classification of humans, animals, and vehicles is proposed in [4]. Magnetometers can be used to detect metallic targets and achieve good accuracy [5]. A target tracking system integrating radio-frequency identification (RFID) with wireless sensor networks (WSNs) is proposed in [6]. Each target must carry an active RFID as the identity classification, which limits its applications in most cases of target tracking. Similarly, the authors in [7] propose a person-tracking algorithm based on luminosity sensors requiring the target must be equipped with a light source, which is also impractical. Different from aforementioned sensors, the authors in [8,9] use sensors providing video images to detect and track targets.

Due to the fact they are absorbed and attenuated significantly in the ocean, many detecting media such as electromagnetic waves and laser waves which can be used to implement terrestrial target tracking methods have poor performance in marine environments [10]. As the only carrier that can propagate over a remote distance in the marine environment, acoustic waves have become the most widely used medium in underwater target tracking. Therefore, the acoustic tracking instrument using acoustic waves for underwater target detection and tracking was born. In this paper, we present a classification criterion for underwater acoustic target tracking algorithms based on the methods used, namely, instrument-assisted methods, mode-based methods, and tracking optimization methods. To allow readers to have a good understanding of this topic, we provide an overview of target tracking theory and introduce three frequently-used acoustic tracking instruments in detail.

This paper concentrates on a comprehensive survey of underwater target tracking algorithms and is organized as follows: Section 2 presents some related surveys and our contributions. In Section 3, we introduce three acoustic tracking instruments, including traditional acoustic sensor arrays (TASAs), acoustic imaging sensors, and underwater wireless sensor networks (UWSNs). The process of underwater target tracking and these main components are clarified in detail in Section 4. Section 5 presents the classification of underwater acoustic target tracking algorithms. Moreover, the discussion, challenges, countermeasures, and lessons learned are presented in Section 6. Finally, we make a conclusion and provide the future work of underwater acoustic target tracking in Section 7.

## 2. Related Surveys and Contributions

In this section, related surveys about target tracking are introduced. In view of the fact that most survey papers are restricted to tracking based on WSNs, the difference between underwater acoustic target tracking and target tracking using WSNs is clarified. Moreover, compared to other surveys, the original contributions of this paper are illustrated.

To our knowledge, there are only a few review articles about target tracking, and they are all focused on target tracking based on WSNs. In [11], the authors are focused on introducing the related knowledge of target tracking for sensor networks, rather than analyzing and comparing different target tracking algorithms. Some other review articles are only centered on one aspect of the tracking algorithms based on WSNs such as energy-efficiency [12], prediction algorithms [13], target recovery techniques [14], and security [15]. As the extension of traditional target tracking in the application of the underwater environment, underwater acoustic target tracking using UWSNs include all the aspect mentioned before. However, due to the special nature of the underwater environment, there exist numerous differences between underwater acoustic target tracking based on UWSNs and target tracking using WSNs.

- *Communication issues*: The underwater channel is a complex channel that is variable over time and space due to the characteristics of the transmission media and the environment [16]. The common communication issues existing in underwater acoustic target tracking are large propagation delay, limited bandwidth and high bit error rate (BER). The speed of acoustic wave in underwater is 1500 m/s, which is five orders of magnitude lower than radio wave transmission. Moreover, the speed is variable with respect to the water temperature, sanity and depth [17]. Therefore, tracking based on distance acquired from time must consider the variation of acoustic speed. The limited bandwidth in underwater acoustic target tracking has motivated researchers to design effective tracking algorithms using quantized measurements. The underwater channel may experience BERs on the order of 10^−2^, resulting in frequent transmission loss. Therefore, robust data fusion techniques are needed in light of the packet loss phenomenon.
- *Real-time*: In underwater tracking, the sensors are usually deployed in 3D-space and drift with the current. The tracking is highly dependent on the accuracy of the sensor positions, which poses more stringent real-time requirements for underwater acoustic tracking algorithms compared with those based on WSNs. The limited bandwidth results in low communication rates which also pose a great challenge for real-time underwater acoustic target tracking.
- *Tracking mode*: Unlike other modems, acoustic modems consume order of tens of Watts of energy during the transmitting process, and tens of milliwatts during receiving processes. The apparent asymmetry in power consumption makes it preferable for underwater acoustic tracking sensors to work in passive mode for energy-efficiency.

Furthermore, the acoustic target tracking not only can be realized by WSNs but also can be dependent on TASA and acoustic imaging sensors which will be introduced in Section 3. However, these surveys about target tracking based on WSNs can provide readers with a comprehensive understanding of the area of target tracking and contrast present tracking methods. Thus, we give a comparison in Table 1. We also compare our papers with other survey papers [11,18] in Table 2, which provides a relatively complete overview of target tracking. With the increased interest in marine resources, the underwater target tracking technology will only get more attention. We believe that a comprehensive survey and detailed introduction of the related knowledge of underwater target tracking algorithms can improve the development of the technology. Our contributions can be summarized as follows:

- Our paper is the first review article centered on underwater target tracking. Many domestic and foreign scholars have thoroughly studied underwater target tracking and numerous papers have been published. Therefore, a survey on underwater target tracking is in need.
- We summarize the abundant related knowledge of underwater acoustic target tracking ranging from the acoustic tracking instrument to the process of tracking, which will provide readers with a comprehensive understanding of this research area.
- We provide an updated literature review of underwater acoustic target tracking algorithms and most of the papers which focus on a study of the underwater target tracking are included. A comprehensive comparison of these algorithms is provided according to the characteristics of the target such as numbers, dimensions, and maneuvering.

The existing review papers concentrate on target tracking algorithms based on WSNs. However, some tracking algorithms cannot be directly applied to underwater target tracking due to the special nature and complexity of the marine environment. Our article is the first paper providing a survey of acoustic tracking algorithms for underwater targets. Meanwhile, tracking instruments are not limited to UWSNs which are variations of WSNs, and TASA and acoustic imaging sensors are also included.

## 3. Knowledge of Acoustic Target Tracking Instruments

To make readers understand the area easily, the basic structure of sensors used for tracking is clarified. Then the most common used acoustic tracking instruments including TASAs, UWSNs, and acoustic imaging sensors are introduced in this section.

As is known to humans, acoustic waves are the only medium which can propagate over a long distance in seawater. As a result, people have been using sound waves to navigate, locate, track and recognize fixed or moving underwater targets. Devices with these functions are acoustic sensors. Compared to the tracking based on WSNs, the underwater acoustic tracking algorithms have to consider some special issues such as communication issues, improvement of real-time and tracking mode. However, the issues in tracking can be solved not only by effective algorithms but also by the tracking instruments. Recently, some researchers are focused on designing acoustic modems for sensors for underwater target tracking to solve the issues incurred by the underwater environment.

**Figure 1:**
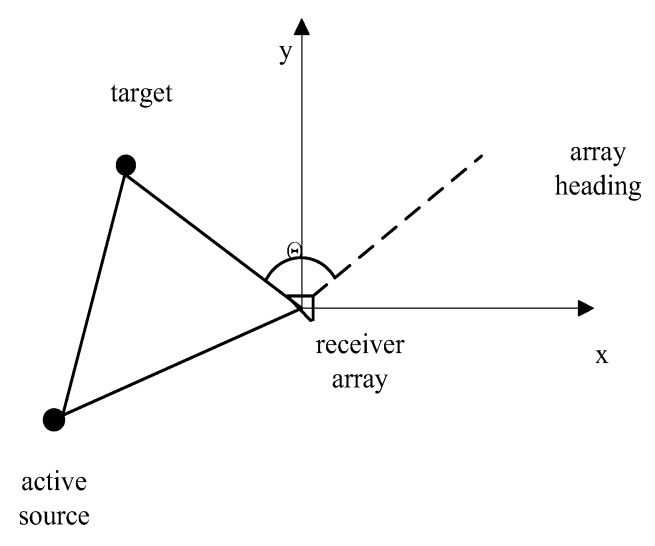
Bistatic source-target-receiver geometry for tracking the interested target.

The authors in [19] provide a comprehensive survey of underwater acoustic modems. The sensors are usually equipped with power, processing and communication units. The most common acoustic sensors have two kinds of use. One is to launch sound waves, and the second is to receive sound waves [20]. The acoustic sensor is usually utilized to transmit and receive sound waves at the same time, while the one especially used just for receiving signals is called a hydrophone. There are varieties of acoustic tracking systems which can be summarized as an active system or a passive system according to the working mode of sensors. Acoustic systems composed of active sensors can be further classified into monostatic systems, where the single sensor serves as a transmitter as well as a receiver; multi-static systems, where the sensor only undertakes the task of transmitting or receiving sound waves. The bistatic system is the simplest type of multi-static systems with a single source-receiver pair [21,22]. Usually, acoustic sources are hull-mounted sensor arrays while the common examples of receivers are towed line arrays as Figure 1 shows [23]. In this section, we will introduce three frequently-used acoustic tracking instruments of underwater target tracking.

### 3.1. TASA

Traditionally, various types of TASAs have been used to complete the underwater target tracking task, for example, the hull-mounted array and towed array [24,25,26]. The hull-mounted array composed of a spherical or cylindrical acoustic sensor array is installed on ships. It is a typical active system which takes the initiative to transmit a certain frequency of sound waves in a row of the array and tracks objects according to the reflection of echoes. The towed array usually made up of a linear array is traditionally dropped into the sea and towed by a submarine, severing as a passive listening sensor which completes the monitoring task completely depending on the radiated noise from the target. 

However, these tracking algorithms based on TASA have fatal flaws in some mission-critical scenarios because they need to be mounted on or towed by a submarine or a ship [27]. The effective tracking area of TASA depends on the trajectory of the tracking platform, which makes intruders easily avoid being detected. In addition, it is impractical and impossible to maintain a 24-h sea patrol, which makes it unsuitable for real-time missions. Among all drawbacks of TASA, the most critical point is that the platform which tows sensor array or on which the array is mounted is going to affect the whole system once an attack or a failure occurs.

### 3.2. UWSNs

**Figure 2:**
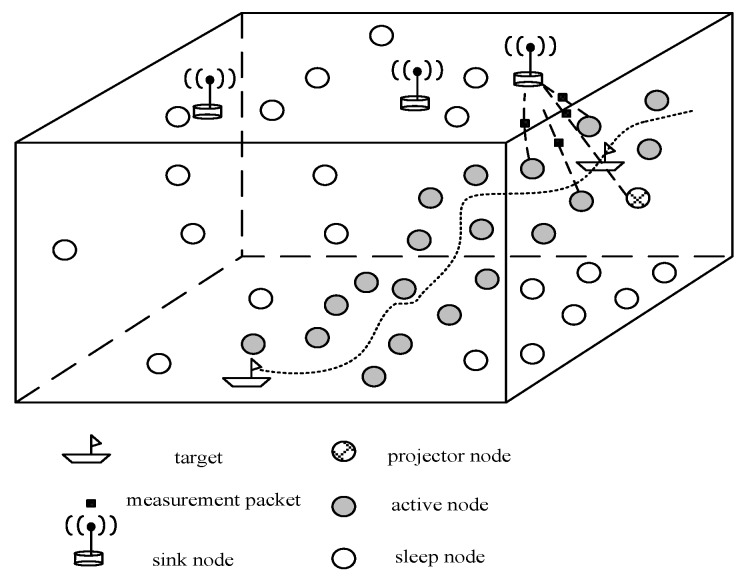
A model of UWSNs.

To address the aforementioned problems, UWSNs, variations of WSNs, are envisioned to enable applications for underwater target tracking. UWSNs have superiority over TASA in low-cost, self-organization, fault tolerance and rapid deployment [28]. UWSNs consist of numerous resource-constrained sensors called sensor nodes. These sensors are equipped with a limited capacity of data processing and communication via acoustic modems [29]. The target tracking problems based on UWSNs can be described as follows. Many homogeneous or heterogeneous sensors are deployed randomly or strategically across the interested area [30]. When the presence of single or multi-target is detected by sampling the sensed signals, these readings from multi-sensors can be used to compute the position of the target and then transmitted to the sink node which has stronger processing ability and generally serves as a gateway connecting the network and application users. After collecting data from sensors in the network, the sink node performs data fusion to provide more accurate position information and estimate the trajectory of the target being tracked [31]. The basic model of UWSNs is shown in Figure 2. Many applications contain the prediction step because the prediction result can be used to reduce energy consumption by activating the set of sensor nodes located in the region where the target is moving [32]. As sensor nodes have the energy restriction, the energy consumption is a key factor for any target tracking algorithms based on UWSNs. To be clear, we identify some components related to underwater target tracking based on UWSNs:

- *Node localization*. Due to the severe attenuation of electromagnetic waves, the global positioning system (GPS) is unable to provide localization service for the underwater sensor nodes. Effective node localization algorithms are in need and the accuracy of the node localization has a significant impact on tracking performance.
- *Node cooperation*. It defines how to communicate between nodes to reduce the amount of message transmitting.
- *Position computation*. Measurements obtained from underwater sensor nodes are severely interrupted by noise which cannot be ignored, therefore, how to use these noisy measurements to obtain the position of the target as close to the true one as possible becomes a challenging issue.
- *Future-position estimation*. Although this process is not necessary for every tracking algorithm, there are many algorithms that take into consideration this information that can be used to save energy consumption of sensor nodes by efficient node scheduling strategies.
- *Energy management*. Because the power of sensor nodes is limited, any tracking algorithms should achieve a good trade-off between tracking accuracy and energy consumption which directly determines the lifetime of the network.

In terrestrial WSNs, as a critical application, target tracking has been well studied and plenty of research results have been published [33,34,35,36]. However, considering the mobility of sensor nodes resulting from ocean current, limited communication bandwidth and the high propagation delay which are three orders of magnitude higher than that in WSNs, these technologies for WSNs cannot be directly applied to underwater target tracking. We must investigate new algorithms suitable for ocean tracking applications. Therefore, in this paper, we also have a brief overview of target tracking algorithms based on UWSNs.

With the development of autonomous underwater vehicles (AUVs), incorporating TASA with UWSNs, a novel variation tracking system of USWNs has been proposed, which is composed of mobile sensor nodes such as the AUVs towing acoustic arrays [37,38]. 

### 3.3. Acoustic Imaging Sensor

Whether based on TASA or UWSNs, these algorithms are non-visual tracking. The development and maturity of underwater acoustic imaging or video processing technology provide an alternative approach for underwater target visual tracking using acoustic imaging sensor. The most widely used acoustic imaging sensor is the looking forward sonar which is usually installed on the front of AUVs. The imaging sensor transmits acoustic signals to the underwater area of interest and processes the received back-scattering echoes. By using acoustic imaging techniques, an initial acoustic image of the real environment is obtained from the image sequences, the underwater target tracking can be realized by determining the target area in each frame image.

In conclusion, there are diversified alternative acoustic tracking instruments for underwater target tracking. However, published survey articles are only centered on WSNs, which is not comprehensive. Therefore, in this paper, a classification, which is based on three commonly used underwater acoustic tracking instruments including the acoustic imaging sensor, TASA, and UWSNs, is proposed in Section 5. 

## 4. Theory of Target Tracking

The general process of target tracking including target detection, position determination, target model construction, state filtering and prediction is introduced in this section. For the integrity of the article, the process of tracking based on images is also covered. 

Target tracking refers to the process of processing the measurements obtained from sensors and maintaining an estimate of the current state of the target. The state mainly contains the position, velocity, and other kinematic components. The measurements refer to the observation information about the target, including direct distance estimation, slope distance, direction of arrival (DOA), received signal strength indication (RSSI), time of arrival (TOA) or time difference of arrival (TDOA), as well as the frequency difference between sensors caused by the Doppler frequency shift. When sensors detect the presence of the target, the observation information can be obtained by sampling the sensed signals (e.g., light, sound, image, or video). Then the position of the target can be computed mathematically by relating this information to the target state. Finally, the tracking process is completed by determining the position of the target at each step. Due to the existence of noise in measurements, an extra component, filtering process should be included to improve the tracking accuracy. Filtering algorithms are also used to predict the position of the target according to the target motion model to accelerate the tracking speed. The process described previously is the general tracking process in non-visual tracking which refers to using TASA or UWSNs.

**Figure 3:**
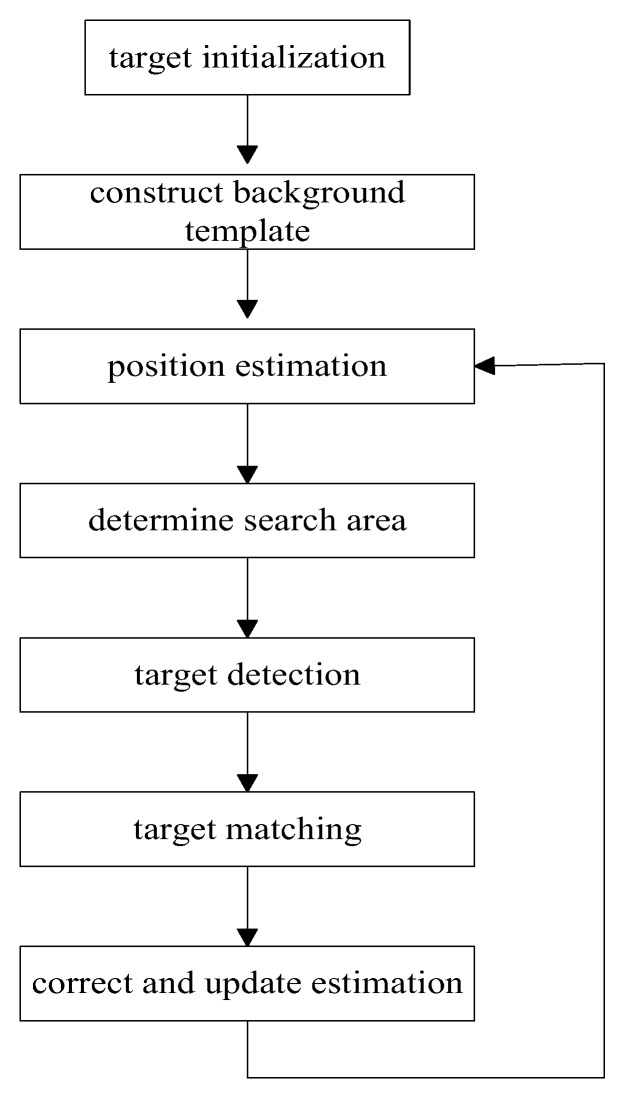
The process of target tracking based on images.

In visual tracking, which refers to using acoustic imaging sensors, the tracking process is a little different. Target tracking based on acoustic imaging sensors involves determining the exact location of the moving target in the next frame quickly by minimizing the search scope of the maneuvering, which can be summarized as shown in Figure 3. Firstly, the initial position of the target is determined by automatic detection or manual selection and the template of the image sequence is established. Secondly, according to the established target motion model and the selected filtering algorithm, the state of the moving target in the next frame such as the speed and position can be estimated, which can be used to determine the search area while avoiding the time consumption of global searching. Then, the target detection is carried out in the search area to obtain the accurate observation value of the moving target. Finally, the estimated value of the target is corrected and the target information is updated to start the next tracking cycle.

In conclusion, the tracking process both in visual tracking and non-visual tracking has some important components such as target detection, position determination, target model construction and state filtering and prediction.

### 4.1. Target Detection

In visual tracking, this process means determining the existence of the moving target in the image sequences and describing the characteristics of the target (such as the position of the target, the size of the external rectangle of the target). Currently, in the field of visual tracking, the commonly used moving target detection algorithms include background subtraction, frame difference, optical flow and mixture of the Gaussian model. Among varieties of target detection algorithms, background subtraction is the most frequently used one. The basic idea of it is to subtract the moving target from the difference image obtained by comparing the background image and the current image [39]. Frame difference is used to separate the moving target by comparing two images in consecutive frames [40]. Optical flow can detect the moving target without prior information about the scene. However, it is not suitable for real-time tracking owing to its relatively high time complexity of computation. A mixture of Gaussian model is aimed at modeling each pixel with a collection of K Gaussian distributions. Then the possibility of each pixel as the background is expressed by the corresponding weight which is calculated through accumulations of the historical input images.

Distinct from the detection based on images, target detection in non-visual tracking is much simpler. An acoustic sensor accuses the detection of the target when the received signal strength exceeds a predefined threshold. It is true that there are many other sophisticated algorithms for detecting targets accurately. More relevant information can be found in other articles.

### 4.2. Position Determination

The process of position determination in visual tracking refers to locate the area of the target in each frame. After a target is correctly detected, the corresponding relationship between adjacent frames should be determined, which is also called target matching. Target matching is completed by the criterion that the center position and size of the same target changes little in consecutive frame images during a small sampling interval [41]. While in non-visual tracking, the position of the target is determined by the location of detecting sensors and the distance of these sensors to the target. The distance information is easily obtained from active sensors. Nevertheless, passive sensors only obtaining bearing information also can be used to determine the position of the target by three bearings from different non-collinear sensors, which is equivalent to using trilateration to compute the position by distance information from three nodes. The position determination algorithms can be classified into range-based and range-free ones. Range-based positioning algorithms determine the distance of a sensor to the target using obtained measurements such as RSSI, TOA, and DOA, then the trilateration, triangulation or multi-lateration are used to estimate the position of the target. Range-free positioning algorithms estimate the position of the target according to the connectivity of tracking networks without range and bearing information. However, the accuracy of positioning is guaranteed by the number of sensing sensors, which means the algorithm is limited for WSNs. The commonly used range-free positioning algorithms are the centroid or weighted centroid algorithms, distance vector hop (DV-hop), approximate point-in-triangulation test (APIT) and convex programming algorithms.

Continuous positioning at the time sequence can also realize tracking tasks, and the corresponding velocity, as well as acceleration, can be obtained according to the position at several sampling instants. However, the state of the target always varies with time and measurements are corrupted by noise seriously, which results in tracking with continuous positioning having poor performance. Therefore, most of tracking algorithms adopt filtering techniques as the technology of state estimation and reducing the noise interference. Position determination algorithms are usually used to obtain the initial state of the target.

### 4.3. Target Model Construction

**Figure 4:**
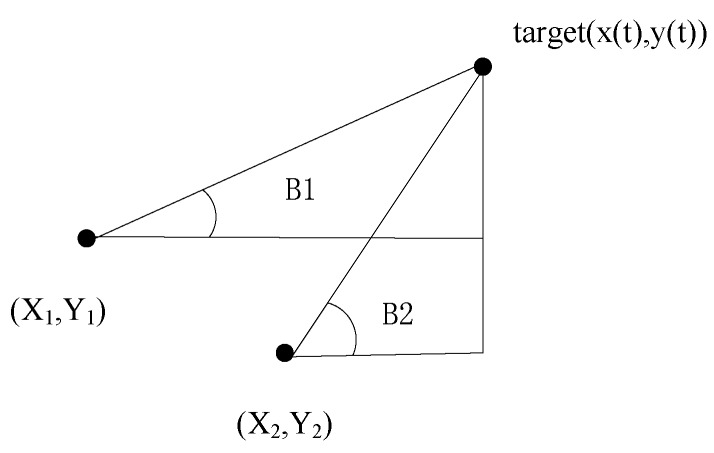
The scheme of obtaining bearing information.

Most of the target tracking problems are investigated based on a model, in other words, based on two descriptions. One is the target behavior represented by the dynamic motion model; the other is the observation of the target called the observation model. The establishment of underwater target dynamic motion model and observation model is the precondition of target tracking [42]. The dynamic motion model and observation model can be collectively referred to as the state space model. The commonly used one is as Equation (1) shows:

(1) $$\{\begin{array}{c} x_{k+1}=f\;(x_{k},w_{k})\\ z_{k}=h\;(x_{k},v_{k}) \end{array}$$

where $x_{k}$, $z_{k}$, $w_{k}$ and $v_{k}$ are the state, measurement, process noise and measurement noise vector at time instant *k* respectively. The transfer function f(⋅) is determined by the target motion, and the measurement function h(⋅) is constructed by the corresponding relationship between measurements obtained from sensors and the state of the target. For example, if the measurement information is the bearings obtained from two passive sensors as Figure 4 shows.

The relationship between the measured bearings and the position of the target can be described as Equation (2) shows:

(2) $$\begin{array}{l} B1=\tan^{}(\frac{y(t)-Y_{1}}{x(t)-X_{1}})\\ B2=\tan^{}(\frac{y(t)-Y_{2}}{x(t)-X_{2}}) \end{array}$$

The measurement function h(⋅) can be represented by:

(3) $$h(x_{k},v_{k})=\left[\begin{array}{c} \tan^{-1}(\frac{y(t)-Y_{1}}{x(t)-X_{1}})\\ \tan^{-1}(\frac{y(t)-Y_{2}}{x(t)-X_{2}}) \end{array}\right]+\left[\begin{array}{c} Bn1\\ Bn2 \end{array}\right]$$

The second term to the right of the Equation (3) represents the measurement noise *ν_k_*. The accuracy of target motion model directly affects the tracking precision. If the model is far different from the actual situation, it may lead to the divergence of the subsequent filtering process [43]. However, it makes no sense to put numerous efforts into modeling as accurately as possible, because the more sophisticated the model is, the more complex it is and the more difficult it is to implement the filtering process. For underwater targets such as submarines, the changing course and speed are the most common maneuver types. Thus, in the marine environment, there are three frequently used target motion models: the constant velocity (CV) model, the constant acceleration (CA) model and the turning model. To provide readers with a preliminary understanding of target motion models, the CV model will be introduced in detail.

In three-dimensional space, the state of a target at time *k* can be represented as $x_{k}=(x,v_{x},y,v_{y},z,v_{z})$, where (x,y,z) is the position vector and $\left(v_{x},v_{y},v_{z}\right)$ is the velocity vector. The state equation is described in discrete time with sampling interval *T* as Equation (4) shows:

(4) $$x_{k+1}=F_{k}x_{k}+Q_{k}w_{k}=\left[\begin{array}{cccccc} 1 & T & 0 & 0 & 0 & 0\\ 0 & 1 & 0 & 0 & 0 & 0\\ 0 & 0 & 1 & T & 0 & 0\\ 0 & 0 & 0 & 1 & 0 & 0\\ 0 & 0 & 0 & 0 & 1 & T\\ 0 & 0 & 0 & 0 & 0 & 1 \end{array}\right]x_{k}+\left[\begin{array}{ccc} \frac{1}{2}T^{2} & 0 & 0\\ T & 0 & 0\\ 0 & \frac{1}{2}T^{2} & 0\\ 0 & T & 0\\ 0 & 0 & \frac{1}{2}T^{2}\\ 0 & 0 & T \end{array}\right]w_{k}$$

where the process noise $w_{k}\in R^{3}$ can be seen as the random acceleration in three dimensions obeying zero-mean Gaussian distribution. If we take the *y* component as an example, the range change in y component and the velocity change in *y* component as Equations (5) and (6) show:

(5) $$y_{k+1}=y_{k}+v_{k,y}\times T+\frac{1}{2}T^{2}\times w_{k,y}$$

(6) $$v_{k+1,y}=v_{k,y}+T\times w_{k,y}$$

### 4.4. State Filtering and Estimation

Target tracking is a typical uncertainty problem. Due to the development of monitoring and anti-surveillance technologies as well as the improvement of target mobility, the problem of uncertainty in target tracking becomes more serious. The source of uncertainty mainly comes from the uncertainty of target motion represented by process noise, the uncertainty of the source of measurements (measurement noise) and the false noise incurred by multi-target and the dense cluttered environment. Therefore, the essence of target tracking is estimating and predicting the state of the target by filtering algorithms to eliminate the uncertainty associated with the target.

According to the dynamic motion model and observation model established, how to choose the appropriate filtering algorithm or improve the existing ones to meet the specific requirements of the underwater target tracking is the key to designing target tracking algorithms. To this end, we will introduce the basic theory of filtering and the commonly used filtering algorithms in underwater target tracking:

*Bayesian filter*: The core design idea of the Bayesian filter is obtaining the posterior probability by using observation data obtained at the current moment to correct the prior probability density function (PDF) [44]. Bayesian theory considers that the posterior PDF obtained by prior PDF and the current system information can better reflect the characteristics of the system. Therefore, the study of the system should be based on the posterior PDF. The Bayesian filter consists of two processes: prediction and update. The goal of prediction is to obtain the prior PDF by the target motion model. The process of update introduces the measurement value obtained from the observation model into the output of the prediction step to correct the probability so that the posterior probability of the target state is acquired.

Let $z_{1:k}=\left\{z_{1},z_{2},\cdots ,z_{k}\right\}$ denote the measurements obtained from time instant 1 to *k*, $p\left(x_{0:k}|z_{1:k}\right)$ denote the posterior PDF before time *k*, and the probability of the initial state of the target is assumed to be known. Since the state vector $x_{k}$ obeys the first-order Markov process generally, the posterior PDF can be recursively obtained using the measurements $z_{1:k}=\left\{z_{1},z_{2},\cdots ,z_{k}\right\}$. Assuming that $p(x_{k-1}|z_{1:k-1})$ has been obtained, the probability of a one-step prediction is described as:

(7) $$p(x_{k}|z_{1:k-1})=\int p(x_{k}|x_{k-1})p(x_{k-1}|z_{1:k-1})dx_{k-1}$$

$p(x_{k}|x_{k-1})$ in Equation (7) represents the state transition probability. The update process utilizes the observation value obtained at time instant k to update the prior probability. The posterior PDF can be derived from Equations (8) and (9):

(8) $$p(x_{k}|y_{1:k})=\frac{p(y_{k}|x_{k})p(x_{k}|y_{1:k-1})}{p(y_{k}|y_{1:k-1})}$$

(9) $$p(y_{k}|y_{1:k-1})=\int p(y_{k}|x_{k})p(x_{k}|y_{1:k-1})dx_{k}$$

where $p(y_{k}|y_{1:k-1})$ denotes a normalized constant, while $p(y_{k}|x_{k})$ represents the likelihood probability.

In linear conditions, the Bayesian filter can acquire the approximately optimal solution, namely the Kalman filter (KF). While in nonlinear conditions, the approximate optimal solution is hard to acquire owing to the infinite dimensional integral calculation. Then some sub-optimal algorithms are proposed such as the extended Kalman filter (EKF), the unscented Kalman filter (UKF) and the particle filter (PF).

*EKF*: This filtering algorithm is developed based on the original KF formulation for nonlinear problems [45]. EKF adopts a Taylor series to linearize the nonlinear part to obtain a sub-optimal estimation. When the maneuvering of the target is not high, EKF can achieve satisfactory results:

(10) $$\begin{array}{l} x(k+1)\approx f(k,\hat{x}(k|k))+f_{x}^{\prime }(k)\left[x(k)-\hat{x}(k|k)\right]+w(k)\\ +\frac{1}{2}\sum_{i=1}^{n}e_{i}{\left[x(k)-\hat{x}(k|k)\right]}^{T}f_{xx}^{\prime }(k)\left[x(k)-\hat{x}(k|k)\right] \end{array}$$

Assuming the estimated state of the target at time *k* is $\hat{x}(k|k)$, the linearization of the state function at time *k* + 1 can be represented as Equation (10) shows. The higher order is omitted here, where $e_{i}$ is the ith standard vector of unit matrix. The summation formula superscripts n represents the dimension of state vector. $f_{x}^{\prime }(k)$ and $f_{xx}^{\prime }(k)$ represents the Jacobian matrix and Hessian matrix of the state transfer vector f at the nearest estimation value of the state respectively. The linearization of the measurement equation at time *k* + 1 is similar, represented as Equation (11) shows. The higher order is also omitted here.

(11) $$\begin{array}{l} z(k+1)\approx h(k+1,\hat{x}(k|k))+h_{x}^{\prime }(k+1)\left[x(k+1)-\hat{x}(k+1|k)\right]+v(k+1)\\ +\frac{1}{2}\sum_{i=1}^{n}e_{i}{\left[x(k+1)-\hat{x}(k+1|k)\right]}^{T}h_{xx}^{\prime }(k+1)\left[x(k+1)-\hat{x}(k+1|k)\right] \end{array}$$

The second and higher order items are omitted in Equations (10) and (11) when the first order EKF is adopted, while the second order items should be kept in the second order EKF. Then the state estimation can be completed according to the basic KF. However, if the nonlinearity of the system is serious, tracking algorithms adopting EKF will result in large errors due to the loss of numerous information in the linearization process using Taylor series.

*UKF*: Based on the unscented transform (UT), UKF approximates the nonlinear distribution using determined sample particles. Different from approximately linearizing the state, UT performs an approximation on the posterior PDF of the state by some determined symmetrical sampling points [46]. The mean and variance of Gaussian PDF can be fully reflected by these sampling points. Therefore, UKF is widely used for solving nonlinear problems with noise in Gaussian distributions. The state prediction and estimation can be carried out by computing the mean and variance of the sampling points undergoing the nonlinear system. The precision of UKF can be compared to the second order EKF because it is not necessary to linearize the nonlinear system which needs complex computation.

*PF*: It is a sub-optimal algorithm of the Bayesian filter based on random sampling [47]. Because it is hard to calculate the posterior PDF in nonlinear or non-Gaussian problems, PF was developed to approximate the posterior PDF based on a set of discrete and random sampling particles. Every particle is equipped with a weight which indicates its quality, and the weighted sum of all particles is the final estimate result. Like the Bayesian filter, PF can be divided into two phases: prediction and update. The prediction phase estimates the state of particles at the next time step according to the settled target dynamic motion model. The update phase recalculates the weights of particles using newly obtained measurements at the current time. As these particles propagate over time, weights of some particles will become increasingly smaller, which is called particle degeneration problem. If the problem is ignored, numerous computations will be wasted on the particles that make few contributions to the estimated result. To address the problem, the re-sampling step, which means updating the set of particles by copying these particles with greater weights and eliminate those with smaller weights, is introduced into PF. The performance of EKF and PF is compared in [48], which shows that PF has superiority in real scenarios. EKF can effectively solve nonlinear problems on the condition that the noise is Gaussian. However, the PDF of the target state cannot be accurately described when the noise is non-Gaussian. PF approximates PDF directly by a set of random sampling points, therefore, it has more precise estimate results compared with the EKF. The results in [44] demonstrate that no other algorithms can perform better than KF when the assumption (with a linear model and Gaussian noise) holds because KF is the optimal solution for that case.

In conclusion, when designing tracking algorithms, researchers should choose appropriate filtering algorithms according to the settled model and application scenarios. Therefore, in Section 5, we take the filtering technique as a subcategory of tracking optimization method. In this subcategory, we introduce and compare tracking algorithms based on different filtering techniques. 

## 5. Classification Underwater Acoustic Target Tracking Algorithms

**Figure 5:**
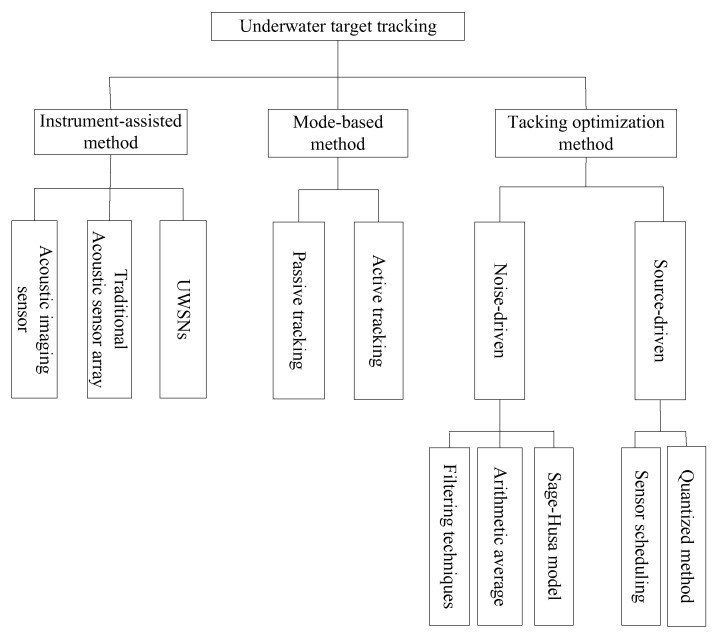
Classification of the underwater target tracking algorithms.

With the development of acoustic technology and tracking instrument, numerous research results have been applied into tracking underwater targets. To evaluate the state-of-the-art underwater acoustic target tracking algorithms, a classification criterion should be proposed and various algorithms should be compared and analyzed within the group with common characteristics. In this section, the classification of underwater acoustic target tracking algorithms is introduced. These algorithms can be classified based on the methods used such as instrument-assisted method, mode-based method, and the tracking optimization method. The categories of commonly used acoustic tracking instruments, namely, the acoustic imaging sensor, TASA, and UWSNs are put into the class called the instrument-assisted method. The mode-based method focuses on how to achieve better tracking results based on passive tracking and active tracking, which are classified according to the working mode of sensors. The tracking optimization method concentrates on improving the tracking accuracy and source consumption. In this paper, filtering techniques, arithmetic average, and the Sage-Husa model are taken as the noise-driven tracking optimization method. The sensor scheduling strategies and quantized methods are taken as the source-driven tracking optimization method. Taxonomy of underwater acoustic target tracking is illustrated in Figure 5.

### 5.1. Classification Based on Instrument-Assisted Method

Instruments used to track underwater targets can be classified as acoustic imaging sensors, TASAs, and UWSNs. Based on the former, the kinematic state (position, velocity, etc.) and other features (size, color, etc.) of the target can be determined by images or video sequences, while the latter can only achieve motion tracking based on the acoustic echo signals. In this section, the algorithms based on different instruments are introduced. These algorithms are compared in the aspect of accuracy, complexity, and cost of tracking based on different instruments, as well as the strength and weakness of the corresponding tracking instrument.

#### 5.1.1. Acoustic Imaging Sensor

With the aid of visual analysis and the development of underwater imaging technologies, it is possible to realize underwater target tracking based on video or image sequences which are obtained by acoustic imaging sensors. Imaging sensors are not attractive compared with other acoustic sensors used in the underwater environment for their limited sensing range and poor visibility. Nevertheless, tracking based on visual data can also be an alternative method, especially in close-range tracking. Many approaches have been taken to overcome the limitations of tracking based on imaging sensors.

In [49], the authors propose a multiple fish tracking system replacing traditional trawl surveys to ensure the safety of the captured fish after sampling. Before tracking, an automatic fish segmentation algorithm including modified Otsu’s thresholding method and histogram back projection procedure is exploited to overcome the problem of low contrast incurred by the fast attenuation and non-uniformity of LED illumination. Then built upon feature-based object matching method, extended Viterbi data association (VDA) is used for multi-target tracking to overcome the challenge of poor motion continuity incurred by a low frame rate (LFR) of capturing results.

The proposed algorithm can be further used for fish abundance analysis and monitoring marine endangered species. However, the drawback of using a single imaging sensor is that it is unable to capture the interested target all the time due to the limited field of view (FOV) and abrupt motion of the target. Researchers in [50] developed a tracking filter that fuses ultrashort-baseline (USBL) measurements and acoustic image measurements to realize reliable underwater target tracking. The filter provides robust tracking performance, even in the case that either USBL or image measurements are not available or faulty. USBL measurements are used for tracking underwater objects with the advantage of easy deployment and relatively long tracking range while providing target position with high variance [51]. The rough target position measurement provided by USBL is used by the imaging sensor to set the region of interest in where the target is located. Then the estimator updates the target position with precise image measurements when the imaging sensor finds the target in its region of interest. In addition to this, the authors in [50] are devoted to adapting the region of interest of the imaging sensor by using the estimated covariance of tracking filter, which effectively avoids false image measurements.

In some dark or murky underwater situations, the images obtained from imaging sensors cannot be used to complete detecting and tracking tasks. The authors in [52] are inspired by the natural phenomenon that neotropical nocturnal freshwater fish generate the electric field to detect the object according to the perturbations in the self-generated electric field. The technique named electrical impedance tomography (EIT), which has been developed for medical applications is extended to the area of underwater tracking to estimate the position and velocity of the target in close range. The simulation results demonstrate that the tracking algorithm based on EIT has lower computation complexity; however, the range between the tracking platform and the target is limited.

Based on the interested target being well detected by some vision-based target detection techniques such as feature-based detection and template-matching detection, the authors in [53] review two typical vision-based tracking techniques: optical flow tracking and mean shift tracking. Optical flow tracking estimates the position by calculating the flow velocity in two consecutive images. However, the performance of optical flow tracking is dependent on the number of reliable corner features tracked from the detected target areas. Mean shift tracking, which is focused on calculating the color histogram in image sequences, locates the position of the interested object according to the matching results of the histogram of the initial image with the later image.

The authors in [53] analyze feasibilities of two typical vision-based tracking algorithms in underwater tracking, while researchers in [49,50] focus on overcoming the drawbacks of visual tracking. The problem of low visibility in underwater images is investigated in [49], while the disadvantage of limited FOV is overcome in [50]. Different from previous articles, in [52], another imaging technique is provided to replace acoustic cameras for nocturnal underwater tracking. In conclusion, tracking algorithms based on image or video sequences are limited to the target in close range. For marine targets which are far from tracking platforms, we should utilize the acoustic waves which can propagate over a long distance in the ocean environment.

#### 5.1.2. TASA

**Figure 6:**
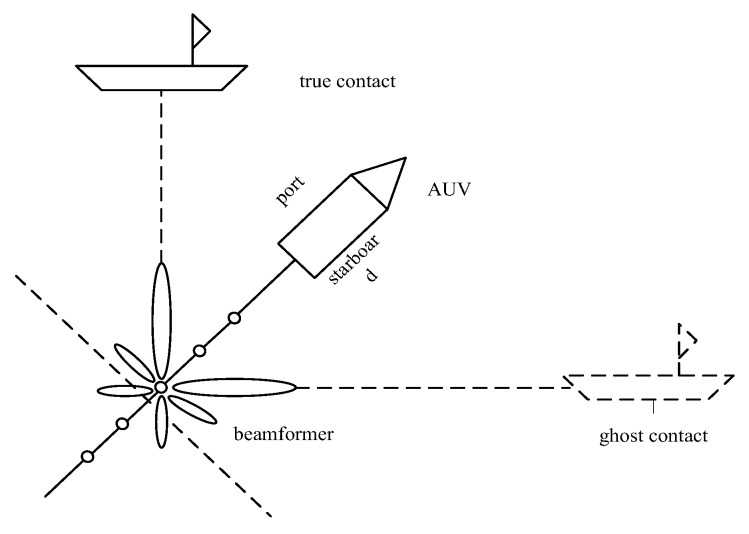
Ambiguity of sensor array.

The sound pressure hydrophone itself has no directivity so it is unable to determine the position of the target on its own. TASA usually uses sound pressure hydrophones forming a linear array model to obtain the bearing information of the target through the method of array signal processing. However, due to the cylindrical symmetry, single line array receivers have the problem of port-starboard ambiguity for tracking, which means generating a ghost track of the target symmetrically displaced with respect to the heading of the array as Figure 6 shows.

Although most algorithms adopt a heuristic approach to address the aforementioned problem [54], the authors in [55] eliminated the port-starboard ambiguity in an optimal way by introducing the ambiguity into the analytical observation model and then the full Bayesian posterior distribution of the target state is derived. Additionally, another novelty of this paper lies in that large amount of AUVs towing acoustic sensor array constitute a novel UWSNs to estimate the target kinematic state at each time scan. During the data fusion process, different from classic “track-to-track” formulation, the fusion and filtering are completed at the same time because the estimated tracks obtained from independent sensors are still dependent due to the common process noise. 

**Figure 7:**
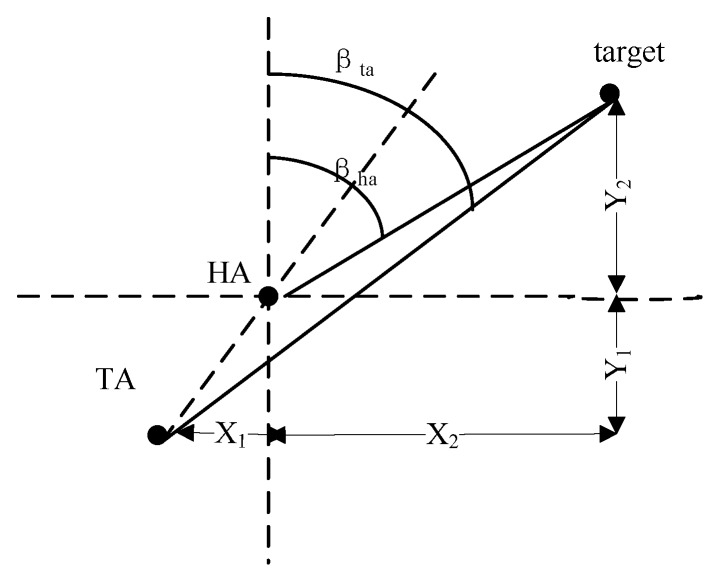
HA and TA tracking sketch.

After obtaining the definite bearing information of the target, the unobservability of the target still exists for the absence of range information especially for the single tracking platform towing or hull mounted the sensor array in passive mode. Some papers focus on designing a proper maneuvering plan for the tracking platform. Nevertheless, in [56], the authors explore the possibilities of completing target tracking using bearings-only measurements without a complex maneuver. In this article, the hull-mounted array (HA) and towed array (TA) are used to obtain bearing measurements from the target as Figure 7 shows. Here *X*_1_ and *Y*_1_ are known by deploying the towed array at a specific place. The central computer adopts data fusion technologies to process the measurements and then the state and trajectory of the target can be estimated by the filtering algorithm. 

Moreover, when a sensor array is used to obtain the bearing information, the quality of tracking is highly dependent on the relative position between the interest target and the array. A target at the broadside brings the highest accurate bearing information, while at the end-fire will cause poor bearing resolutions. Therefore, the authors in [38] propose an adaptive control strategy for the vessel towing the array to keep the target at the broadside as much as possible by the acquired measurement information.

The matched-field processing (MFP) method is also can be applied to the area of underwater tracking. MFP determines the position of a target based on matching the complex acoustic field observed by a sensor array with the replica of an acoustic field computed numerically according to the acoustic prorogation model [57]. The most challenge problem in MFP is the environmental mismatch. In [58], a Bayesian approach is developed for tracking multiple acoustic sources using MFP. When the properties of the ocean environment are not well-known, the proposed algorithm shows great performance. Firstly, the problem of environmental mismatch is formulated in terms of PDF over the model parameters. Then, PDF can be marginalized over all parameters to obtain the probability ambiguity surfaces (PAS) which is the probability distributions over source range and depth. Tracking estimates can be extracted from the PAS by the Viterbi algorithm.

In this section, different tracking algorithms based on TASA are reviewed. The problem of estimating the state of a target is assumed to be two-dimensional in [55], which is not practical in underwater target tracking. Moreover, the SNR is assumed to be uniform and constant in the monitoring region, which is also invalid due to the interference in the multi-path environment, especially in the ocean environment. Compared with [55], the ambiguity is assumed to have been removed and the accurate bearing information can be acquired in [56]. It is worth highlighting in this paper that the problem of unobservability is eliminated by an innovative means, using two arrays cooperatively obtaining bearings from the target. However, the algorithm has the limitation of detecting accuracy when the noise of measurements exceeds three degrees. What’s more, the communication channel is assumed ideal during the period of simulation, which is difficult to meet in a practical implementation. The authors in [38] are aimed at investigating the autonomous decisions for moving tracking platform to improve tracking performance. The simulation results show that the algorithm significantly minimizes the estimation error of the target position and provides a method of maintaining the track. Different from previous papers, the authors in [58] using MFP for tracking focus on overcoming the problem of tracking error incurred by environment uncertainty; however, this algorithm is only appropriate for tracking the target emitting signals with a certain strength, which restricts the application of it in underwater target tracking.

#### 5.1.3. UWSNs

The development of microelectromechanical devices and wireless technologies leads to the emergence of inexpensive small sensors equipped with sensing, processing and communication capabilities [59]. UWSNs integrate numerous sensors deployed randomly or strategically in the monitoring area to complete underwater target tracking tasks. Compared with TASA, the collaborative nature of UWSNs brings several advantages such as self-organization, rapid development and inherent intelligent processing capability [60]. However, there are also many constraints for UWSNs. To ensure the tracking accuracy, it is desired to get as many measurements as possible from the sensors. If let all sensors work in detecting mode, for the limited energy and the fact that it is impractical to recharge the battery on each sensor, the lifetime of the USWN will be much shorter. Therefore, most of the underwater tracking algorithms based on UWSNs are focused on the trade-off between tracking accuracy and energy consumption.

In [61], a tracking algorithm based on distributed UWSNs is proposed to increase the energy efficiency of each sensor node. This algorithm includes a wake-up/sleep (Wus) and valid measurement selecting (VMS) scheme. The estimated position of the target provided by KF is used to wake up sensors which could detect the target. The scheme of VMS determines the valid sensors which can detect the target among activated sensors and passes their measurements to the processing node to update the state of the target. Based on the previous work, the authors further extend the tracking algorithm to track maneuvering target based on UWSNs by combining the interacting multiple models (IMM) with energy-efficient schemes Wus and VMS [62]. 

A distributed smart sensor scheduling method is proposed to save energy at the level of sensors in [63]. Each node is modeled as a probabilistic finite state automaton (PFSA). According to the local information, the sensor updates the probability of PFSA to switch the working mode between sleeping, listening, low sensing and high-power sensing to minimize the energy consumption. As the energy model shown in [64], compared with the energy of the sensor node consumed during the process of obtaining and processing measurements, the communication with other sensors or the sink node dominates the whole energy consumption. Therefore, there is a vast literature focused on reducing the amount of communication to save energy. In [65], by cutting down the useless information communication between local sensors and the fusion center, the energy consumption has been dramatically decreased. The algorithm requires that the distributed sensor in UWSNs determines whether sending its measurement to the fusion center according to the value of measurement residual which indicates the new measurement containing how much information to update the state of the target. However, the threshold of measurement residual has a great effect on the tracking performance and this paper does not provide methods about how to determine the threshold.

Among these energy-efficient tracking algorithms, the common characteristic is that not all measurements obtained in UWSNs are adopted or not all sensors work in active mode, which will surely reduce the accuracy of tracking. In [65], the authors design a scheme of selecting the obtained measurements to send, thereby saving energy by cutting down the amount of communication. To ensure the tracking accuracy, corresponding artificial measurements are generated in the fusion center to compensate for the unsent measurement. In [66], the authors propose an adaptive sensor scheduling strategy for underwater target tracking based on UWSNs, which is named accuracy-stressed and energy-concerned (AnE). The strategy is designed for saving energy based on guaranteeing tracking accuracy. According to the trilateration, four sensor nodes can determine the position of the target. To improve tracking accuracy, the best sensor group consisting of four sensors is picked out at each time step from the candidate sensors based on the criterion of predicted tracking accuracy generated by combining IMM with EKF. Then one member of the best sensor group which can minimize the communication burden is selected as the fusion center to reduce energy consumption. Additionally, the sampling interval is increased when the tracking accuracy is satisfactory, which can further increase the energy efficiency. Compared with other energy-efficient tracking algorithms, this paper keeps the tracking accuracy first, nevertheless, the selection of best sensor group will incur excessive computation cost when the number of the candidate sensors is large.

Since UWSNs consist of numerous sensors, the topology of these sensors will also have a great effect on the tracking performance [67]. Inadequate sensor configuration will yield significant tracking errors no matter what tracking algorithm is utilized. Therefore, there are some papers that consider the configuration of sensor deployment to improve the tracking performance. The authors in [68] investigate the problem of optimal sensor deployment for underwater target tracking using range-only measurements. Based on the conclusion obtained from [69] that the sensor configuration derived from the Fisher information matrix (FIM) of a 3-D scenario can estimate the position of the target accurately by an unbiased estimator, the authors in [68] compute the optimal geometric sensor formation of UWSNs with arbitrary number of sensors by maximizing the FIM to obtain as much range-related information as possible.

Recently, the complex network theory is employed to solve the topology control problem of UWSNs [70]. Based on the concept that UWSNs can be abstracted and analyzed as a scale-free complex network, the authors in [71] provide a mathematical model embodying the most typical characteristics of UWSNs such as large propagation delay, energy consumption, and signal irregularity. Firstly, a mode named edges constructed model (ECM) is used to generate the initial topology. Then a topology control strategy based on the complex network theory (TCSCN) is proposed to evolve the initial topology to a double clustering structure which can effectively ensure achieving (1, ξ)-Coverage and (1, ξ)-Connectivity while optimizing the propagation delay and energy consumption. 

Previous topology control strategies are focused on UWSNs with stationary nodes, while for mobile networks, the problem is converted into optimizing the trajectories of sensors. The authors in [72] investigate how to optimize the trajectory of sensors in the mobile wireless network and an effective approach named optimal control (OC) is presented. An integral objective function representing the track coverage of the sensor work is obtained using geometric transversals. On this basis, the problem of determining the trajectory that maximizes track coverage and minimizes energy consumption can be transferred into a mathematical problem which can be solved by OC. By implementing this approach along with two traditional methods on three networks with different sizes, the authors demonstrate that OC can significantly improve the performance of the sensor work compared with average coverage and path planning method. 

The success of [72] lies in the fact that the authors obtain suitable objective functions and system models, which makes it possible to apply OC to mobile sensor networks. However, the result is obtained from simulations, which lacks credibility. Moreover, the tracking performance of two algorithms proposed in [71,72] for the optimization of the structure of UWSNs has not been presented. The authors in [61,62,63,65,66] focus on the energy consumption of the tracking based on UWSNs. The consumption is reduced by turning off unnecessary sensors in former three papers, while by cutting needless communication between sensors in the latter two papers. Energy consumption and tracking accuracy are considered simultaneously in [65,66]; however, the tracking accuracy is kept first in [66]. The most difference in [67] is considering the quantized measurements.

In this section, algorithms based on different tracking instruments are reviewed. These algorithms are all oriented at or devoted to solving the inherent flaws of tracking instruments. For example, UWSNs have the limited resources, therefore, considerable papers are focused on the limitation. To make readers easier to select the appropriate tracking instrument, we compare these papers in the aspect of precision, complexity, and cost of tracking based on different instruments, as well as the strength and weakness of the corresponding tracking instrument in Table 3.

### 5.2. Classification Based on Mode-Based Method

According to the working mode of tracking sensors, tracking algorithms can be categorized into passive tracking and activity tracking. In the former, the tracker can only estimate the state of the target by the signal directly emitted from the target. In general, the obtained measurement is the relative bearing of the target to the tracker. While in the latter, the extra range information about the target can be acquired by the time of arrival (TOA) or time difference of arrival (TDOA) because these echoes reflected from the target are produced by the tracker. In other words, active tracking contains both transmitters and receivers while the passive tracking only includes receivers. In this section, algorithms based on different tracking mode are introduce and compared in tracking precision. The technology and measurements adopted in every algorithm are also summarized.

#### 5.2.1. Passive Tracking

The problem of tracking using passive measurements can be formulated as bearings-only tracking (BOT) which is a technology of determining the state of a target solely through measurements obtained from the signals originated from the target. Due to the inherent nonlinearity between the passive measurements and the state of the target, the design of a reliable and robust algorithm for BOT is still a challenge.

In [73], since the pair of two sensors located on the towed array can give a rough estimate of the position of the target through the bearing measurement obtained individually as Figure 4 shows, the different measurements obtained with multiple sensor pairs of the towed array are given to multiple filters to obtain multiple state estimates and corresponding covariance matrices. Then these estimates are integrated by least square estimation (LSE) to obtain the final fusion result.

**Figure 8:**
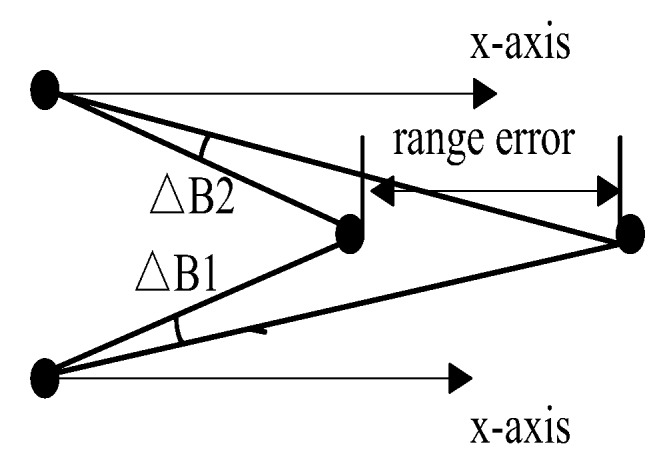
Geometry to illustrate the effect of bearing error.

The passively acquired bearing measurements of the targets are often corrupted by noise. If these bearing or azimuth measurements are directly used to compute the position of the target [73], even small errors in angle can result in huge position deviation. This is apparent from Figure 8. A novel tracking algorithm dealing with the BOT problem is proposed in [74], which reduce tracking errors by the pre-processing process of the obtained bearings. The preprocessing technique efficiently reduces the variance of the noise present at the bearing measurements by taking an average of the present and projected the previous measurement since the natural noise is generally unbiased. The proposed pre-processing technique makes it possible to achieve satisfactory tracking performance using passive measurements in underwater scenarios with high environmental noise.

The tracking performance can be improved by a higher order of pre-processor; however, it is at the cost of bringing the extra computation complexity and time. The optimal number of the order is left for future work.

The BOT has the problem of unobservability before the single observing platform making a maneuvering. Like the work in [56] which eliminates the unobservability by adding another platform, the authors in [75] add another stationary sensor providing range information by the time-delay of the passively acquired echoes emitting from the target. With bearings measured by a sonar and the time-delay between the sonar and a sensor, the tracking performance using passive measurements has been significantly improved. Compared with the system in [56] with a towed array, the system is much easier to design and realize in [75].

The BOT problem existing in most passive tracking scenarios poses a great challenge in multi-target tracking and trace across processing [76]. To address the multi-trace crossing problem, the authors in [77] innovatively propose the single double side constant false alarm rate (SD-CFAR) to detect possible targets from the bearing-time measurements. Then the multi-track gate method is adopted to choose proper measurements belonging to the trace of each target at the current time. Then the auto-regression (AR) model is exploited to solve the problem of trace interruption due to the missing of tracking points incurred by detection error or trace crossing. If one measurement is available for a trace, this trace can be updated by the measurement using KF. Otherwise, the AR model is carried out to predict the missing points, and the number of missing points determines either the trace to be updated or terminated. Similarly, the authors in [78] propose the probabilistic multi-hypothesis tracker (PMHT) for BOT problem to realize multi-sensor-multi-target tracking. The algorithm solves the data association and target estimation using the expectation-maximization (EM) algorithm and KF respectively. This algorithm performs very well in the clutter environment; however, the performance of it is dependent on the accuracy of the initial state estimation about the target.

Recently, measurements in passive tracking are not limited to bearing measurements anymore, and it can contain the time-delay measurement obtained by hearing the emitted signals arriving at different sensors as [75] shows. However, the relationship between the target state and time-delay measurements is much complicated than the relationship between bearing measurements. A network consisting of underwater gliders is proposed in [37], where an array of two sensors embedded on each glider provides the passive measurements of time-delay for tracking purpose. To solve the previously mentioned problem, an adaptive compressive sensing and processing (ADCSP) method is employed to ensure the accuracy of tracking while reducing the measurement sampling rate and producing a low volume of measurement statistics transmitted to the fusion center. The introduction of the compressive sensing and processing method relaxes the requirement for the length of the sensor array and hardware hosted on the glider, which is the most significant contribution of this paper.

**Figure 9:**
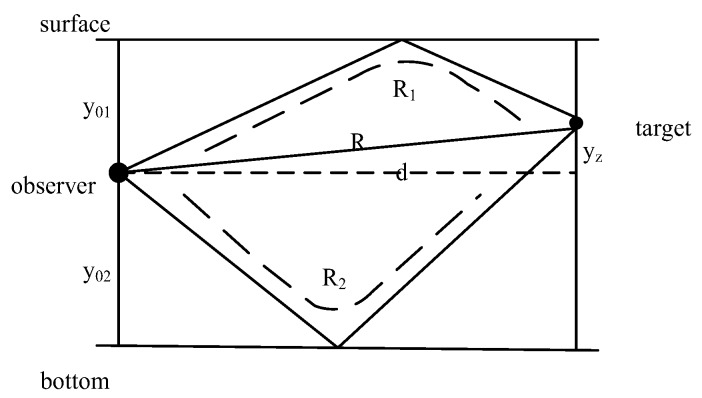
The tracking model of Hassan.

Unknown environmental factors such as water depth, salinity, and currents pose great challenges for underwater target tracking [79]. Furthermore, plenty of undetermined noise in marine environments also causes the time-delay passive measurements being severely corrupted. As a result, a simple model is unable to address the underwater tracking problem. In [80], the tracking model of Hassan is extended to modeling for the bottom and surface reflection of sound in ocean environments as Figure 9 shows. Then the time-delay between the bottoms reflected signal and the direct one as well as the time-delay between the surface reflected signal and the direct one can be used to calculate the distance of the target to the observer. To improve tracking performance, the tracking model is developed by considering the sound speed variation resulted from the effect of water depth.

The extra range information about the target is obtained by using multiple detecting platforms in [37,75] or utilizing the reflection in [80], which makes the tracking much easier than that in [56,73,77,78] only having the DOA measurements. Compared with other passive tracking algorithms, the method proposed in [77,78] can be used for tracking multi-target. Reference [79] is one of the few tracking papers which attach importance to and propose a corresponding resolution for the effect of unknown environmental factors on underwater target tracking.

#### 5.2.2. Active Tracking

The tracking algorithms based on active tracking are aimed at estimating the state of the target according to the bearings and range information provided by the echoes from the target, and the echoes are originated from the system itself. Therefore, systems of active tracking contain transmitters and receivers. However, systems containing active sources have poor governance in anti-submarine warfare (ASW) due to the concealment. The position of the target is also easily obtained because of the available range information provided by active measurements. Therefore, the tracking algorithm based on active measurements is not the attractive research center of the underwater tracking area, especially in their applications in ASW. There are few papers researching the active tracking.

In [38], a multi-static system, where a sonar source (transmitter) located on a stationary buoy or ship emits a ping and the echoes reflected from the target are collected by multiple receivers (AUVs towing sensor array), is used to generate bearing-range contacts fed into the tracker to produce tracks.

In [81], a bistatic system is used to obtain active measurements of the target. The transmitter sends the signal with the certain strength to the target, then the target reflects the signal to the receiver. The receiver estimates the relative range between it and the target according to the change of the strength. The mathematical model is expressed as follows:

(12) $$SE=SL-TL_{1}-TL_{2}+TS-L_{e}-Nrd_{n}$$

In Equation (12), *SE* and *SL* denote the strength of the signal received by the receiver and that transmitted by the transmitter respectively. *L_e_* represents the level of noise out of beam-former and *Nrd_n_* is related to the ability of receivers to recognize noise. *TL*_1_ and *TL*_2_ represent the transmission loss from the transmitter to the target and that from the target to the receiver respectively. The range information is implicit in the transmission loss *TL*_1_ and *TL*_2_:

(13) $$TL_{1}=20\log(R_{tt})$$

(14) $$TL_{2}=20\log(R_{tr})$$

$R_{tt}$ in Equation (13) is the relative range between the transmitter and the target, and $R_{tr}$ in Equation (14) is that between the target and the receiver. The range information can be obtained according to the SE using the figure of merit (FOM). In other words, the point of SE curve crossing with the FOM indicates the estimated range of the target with respect to the receiver. However, the SE is acquired with some degree of noise, which results in the tracking error that cannot be ignored. To this end, the fuzzy c-means method (FCM) and KF are employed to classify the source of noise and compensate the tracking error respectively.

In [82], the authors propose a 3-D underwater target tracking (3DUT) algorithm based on UWSNs. By sending acoustic pulses actively, the distance from the target to the sensor node is estimated and calculated according to the time-delay measurements. Then the location of the target is determined at the sink node using trilateration. To achieve tracking, the process of calculating the location of the target should be performed continuously. In this paper, each sensor node in UWSNs is equipped with the capabilities of actively sending pulses and passively listening to the echoes from the target. Among activated sensors which have the probability of detecting the targets, only one sensor named as a projector node is selected to transmit pulses periodically during the tracking process. Other activated sensors only receiving echoes are called hydrophone nodes. Different from that the active source in [38,81] is stationary, the projector node in this paper serving as the active source can be changed with the trajectory of the target.

Like [42], the authors in [83] also use trilateration to calculate the position of the target. However, because of underwater noise, the position information obtained from the trilateration is not accurate and even unavailable for the equation of trilateration having no resolution. To this end, the authors in [83] propose a consensus estimation algorithm based on 3DUTT, which makes use of the fusion concept to improve the tracking accuracy. Compared with the scenario that there is only one designated projector node actively transmitting pulses in [42], each hydrophone sensor in this paper can send acoustic wave after detecting the presence of the target, which means activated sensors can directly obtain relative distances to the target. For the three-dimensional underwater target, every four non-coplanar sensors forming a cluster can determine the position of it. To save energy consumption, the four sensors broadcast and receive the remaining energy from each other. Then the node containing the maximum remaining energy is selected as the cluster head to calculate the rough location information of the target. The consensus estimation algorithm based on 3DUTT is aimed to fuse this rough information to reduce the negative effect of noise on the final tracking result.

References [38,81] share in common that the active source is stationary. However, this will bring high tracking error owing to the severe propagation loss in an underwater environment, especially in the case that the target is far from the active source. Different from previous works, the transmitter in [82,83] can be changed with the trajectory of the target. During tracking process, there is only one transmitter in [82], while there are multiple active sources in [83] which will result in extra energy consumption compared with that of [82]. 

In this section, various algorithms based on tracking mode (passive and active) are introduced in detail. Now, we compare them in the aspect of tracking precision in Table 4. To make readers know the algorithm proposed in each paper quickly, we also list the main tracking technologies and the types of measurements used in each paper.

### 5.3. Classification Based on Tracking Optimization Method

According to the tracking instrument and tracking mode, researchers have proposed numerous algorithms to improve the tracking accuracy and stability. However, other optimization methods have also been investigated for further improvement. Here, we take the filtering techniques, arithmetic average, and Sage-Husa model as the tracking optimization methods driven by the noise. The filtering techniques such as KF, EKF, UKF, and PF are used to estimate the state of the target and decrease the interference of noise on the tracking performance. That’s why we take the filtering techniques as the tracking optimization methods. The sensor scheduling strategies and quantized methods are the subcategories of the tracking optimization methods driven by the limited source.

#### 5.3.1. Noise-Driven Optimization Methods

The filter algorithm plays an important role in the tracking process for reducing the noise interference. In most tracking problems, the target is usually described by the state space model which includes the state and observation equation. Assuming $Z^{k}=\{z_{1},z_{2},\ldots ,z_{k}\}$ is all measurements up to time *k*. The estimation of $x_{k}$ is called state filter, and the estimation of $x_{k+l}$, l>0 is named as state prediction. In this part, we classify the algorithms based on using different filters. In the existing literature, underwater target tracking algorithms are usually based on KF, EKF, UKF, and PF. Because EKF and UKF are variations of the KF for nonlinear problems, we classify the tracking algorithms into that based on KF and PF. Compared to terrestrial tracking, underwater target tracking is in face of more serious noise interference. Therefore, some data pre-processing techniques such as arithmetic average and Sage-Husa model is required. 

##### Filtering Techniques

*KF*: It is a recursive estimation method based on the state space model, which is aimed at producing estimates with high accuracy by a set of low-level and redundant measurements. However, KF is only suitable for tracking the target with linear motion and moving in the environment white Gaussian noise. Therefore, EKF and UKF, variations of KF, are developed for solving the nonlinear problem.

The algorithm named 3DUTT is employed to determine the location of the target with the trilateration in [82]. However, due to the lack of a prediction step, the tracking accuracy is not satisfactory. Inspired by this, the authors in [84] propose an adaptive underwater target tracking algorithm based on KF for UWSNs, where the output of trilateration is inserted into KF as the measuring model to produce final results with high accuracy.

Since bearing measurements have a nonlinear relationship with the state of the target, vast literature considers adopting EKF and PF in some bearing-only tracking cases [84,85,86]. However, in practice, underwater object maneuvering can be described as a simple linear model because the angle changes little during the sampling interval for the limited speed of the target compared with the long distance between objects and the receivers. Based on this concept, the authors in [77] use KF, which is an optimal estimation algorithm with lower computation complexity compared with EKF, UKF, and PF, to track the underwater target.

The existing range-based tracking algorithms usually locate the target by the distance to some sensing nodes, and the distance is obtained by multiplying the speed of the acoustic wave by the traveling time. However, the sound speed is not constant in underwater environments, which results in the range-based tracking algorithms having poor tracking performance. To address the aforementioned problem, an isogradient sound speed profile (SSP) is adopted in [87] to formulate the range-based localization problem with the time-based problem. Then the localization is completed by multi-lateration and EKF is utilized to track the moving target in a recursive manner. However, this paper does not take the energy consumption of UWSNs into consideration, which leads to the limitations for its practical applications.

An adaptive KF is proposed for tracking underwater maneuvering target in [88]. The algorithm uses another KF to estimate the process noise variance of the main KF, which effectively improves the tracking accuracy and reduces the response time of the filter for maneuvering behaviors of the target. Nevertheless, the algorithm ignores the energy consumption of UWSNs and is only validated in the case of tracking the 2-D target, which is impractical for underwater target tracking. Similarly, in [60], KF is applied in a distributed architecture to estimate the position of a single target moving through UWSNs and improve energy efficiency. However, only the case of tracking 2-D targets is investigated.

Based on the concept that the collective estimation is better than the single estimation, a novel tracking approach based on UKF is proposed for BOT problem in [73], which is named as integrated unscented Kalman filter (IUKF). In the tracking process, each sensor pair consisting of two sensors of the towed array provides bearing measurements and the corresponding covariance matrix which are given to the UKF to obtain a rough state estimation. Then these several rough state estimations are fused by using LSE to get the final more accurate state estimation. The simulation result demonstrates that the integration technique has better performance over the traditional one in terms of the estimation error and convergence time.

Considering the complexity of PF, the authors in [78] address the BOT problem of tracking multi-target by exploiting EKF and UKF combined with PMHT, which is called PMHTe and PMHTu respectively. Both algorithms show great performance in the environment with low measurement noise while the PMHTu dominates in that with high measurement noise.

*PF*: A two-stage PF-based technique is proposed for tracking multi-target based on passive tracking in [89]. Compared with the traditional PF technique sampling from only a single important density, a variation filter named the Mixture PF samples from a mixture of important densities containing the prior and the observation likelihood. To solve the problem of tracking an unknown number of multiple targets, two Mixture PFs are employed. The role of the first filter is target detection. Then the particles corresponding to the detected targets are clustered using one density-based clustering technique. The second filter tracks the detected target by taking these different clusters as the input.

For the problem of tracking targets with weak signals, a tracking algorithm is proposed for underwater targets by combining PF with track-before-detect which is named as PF-TBD in [90]. The method is efficient for tracking underwater targets with low signal-to-noise ratio (SNR) by adding an existing variable to the state of particles for existence probability estimation. Different from previous works of the PF-TBD, the algorithm in this paper takes data collected by a vertical line array as the measurements, which can simplify the probability model and have better performance than traditional MFP whatever in cases with low or high SNR.

There are some papers proposing PF-based tracking algorithms for WSNs [91,92]. The two papers have common in adopting distributive PF for the sensing nodes in WSNs to track the target. Similarly, the authors in [93] propose two tracking algorithms based on a distributed PF for cluster-based UWSNs. Both run local PF sequentially at each cluster along the trajectory of the moving target. However, two tracking algorithms are different in the scheme of selecting measurements to keep a balance between the tracking accuracy and the cost including the communication cost and the energy cost.

Tracking underwater targets with video sequences is a great challenge due to the complex background. A new algorithm combining the real-time of Mean Shift and the robustness of PF is proposed in [94]. The tracking scene in this paper is the swimming pool, for which the color histogram is suitable as the target model because of the stability of the color feature in scale, rotation, and translation. Therefore, the underwater target model is based on RGB histogram according to the kernel function and Mean Shift. Then the tracking process is completed by PF. Owing to the introduction of Mean Shift, the distribution of particles can quickly get closer to the true state of the target. Therefore, the state can be represented by fewer particles, which can further reduce the tracking time to ensure real-time tracking.

From the above introduction of various tracking algorithms based on different filtering selections, we know that the EKF, UKF, and PF are commonly used filters in underwater target tracking because the target model of underwater targets is usually nonlinear and the noise is non-Gaussian. By analyzing data collected from the looking forward sonar, the authors in [95] compare the tracking performance of EKF, UKF, and PF for the underwater target with different motion models such as CA, CV, and the variable speed linear model. Except the last model is maneuvering model, the other two target models are non-maneuvering models. In summary, all EKF, UKF, PF based methods can provide accurate tracking results for the linear model. However, for nonlinear stochastic movement and systems with non-Gaussian noise, the effectiveness of methods based on EKF cannot be guaranteed. When systems are nonlinear and with non-Gaussian noise, the method based on PF has superiority over that base on UKF. 

##### Arithmetic Average

**Figure 10:**
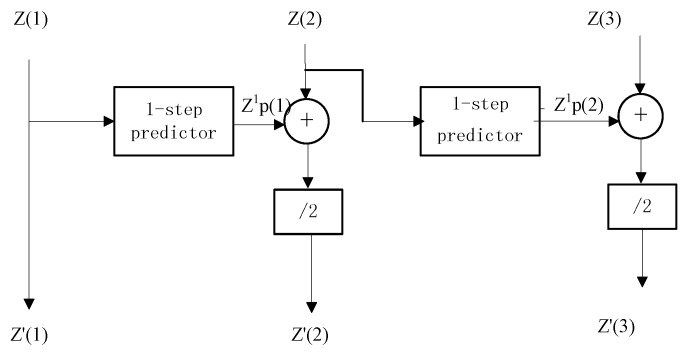
The 1-order pre-processor.

Because of the complexity of the marine environment, the acquired measurements are usually corrupted seriously by noise, and the measuring error may result in large tracking error even employing filtering techniques. In [74], a novel tracking algorithm is proposed to address the problem of noise interference. The measuring error in bearings is removed by pre-processing progress using arithmetic average. The pre-processing technique efficiently reduces the variance of the noise present in the bearing measurements by taking an average of the present and the projected previous measurements since the natural noise is generally unbiased. To be clear, the example of 2-order pre-processor is shown in Figure 10.

The detailed computation process can be seen in Equation (15), where *Z*(1), *Z*(2) and *Z*(3) denote the measurements acquired at the time instant 1, 2 and 3 respectively. The corresponding pre-processing result is represented by *Z*^′^(1), *Z*^′^(2) and *Z*^′^(3). *Z*^1^*p*(1) and *Z*^1^*p*(2) denote the 1-step predictor measurement of *Z*(1) and *Z*(2) respectively:

(15) $$\begin{array}{l} Z{}^{\prime }(1)=Z(1)\\ Z{}^{\prime }(2)=\frac{1}{2}\left[Z(2)+Z^{1}p(1)\right]\\ Z{}^{\prime }(3)=\frac{1}{2}\left[Z(3)+Z^{1}p(2)\right]\\ Z{}^{\prime }(4)=\frac{1}{2}\left[Z(4)+Z^{1}p(3)\right] \end{array}$$

The 1-step predictor measurement *Z*^1^*p*(1) and *Z*^1^*p*(2) in Equation (15) can be computed by taking an average of one-step differences of previous *N* measurements as follows:

(16) $$\begin{array}{l} Z^{1}p(1)=\frac{1}{N}\sum_{i=2-N}^{1}\left[Z(i)-Z(i-1)\right]\\ Z^{1}p(2)=\frac{1}{N}\sum_{i=3-N}^{2}\left[Z(i)-Z(i-1)\right] \end{array}$$

where the number of *N* can be chosen according to the real application. In Equation (16), the negative index measurements refer to the measurements stored before the start of the algorithm.

##### Sage-Husa Model

Traditional arithmetic average as previously mentioned is not applied to the time-varying system because the coefficient is same. In [81], a statistical estimation model named as Sage-Husa is employed to estimate the measured noise online. The Sage-Husa model estimates the measurement noise and process noise from the statistics of obtained measurements based on the concept that the new data should be attached more importance to while the old one should be weakened gradually. The model is demonstrated to reduce the tracking error significantly and make the following filter process converge with a quick speed, especially when the interference noise is unknown. Due to the dynamic estimation about the noise using the Sage-Husa model, the following filter can be adaptive to any tracking environments.

#### 5.3.2. Source-Driven Optimization Methods

In general, underwater target tracking is realized by multiple sensors in which case the communication issues are inevitable. Due to the limited communication bandwidth in underwater channel, the amount of transmission message is restricted. The most effective way to reduce the length of communication message is quantization, which means compressing the raw measurements or state estimation into several bits of data. However, the quantized procedure degrades the performance of tracking accuracy for the loss of information. Recently, some studies are focused on addressing the problem of improving tracking performance with optimal quantization. The acoustic sensors used for underwater target tracking are equipped with limited energy and communication capability, which motivated the presence of effective sensor scheduling strategies. Tracking optimization means not only improving tracking accuracy but also considering the source constraints. Therefore, source-driven optimization methods including sensor scheduling strategies and quantized methods are presented here.

##### Sensor Scheduling Strategies

Due to the limited energy source of sensors, effective sensor scheduling strategies are important for robust tracking by avoiding node failure incurred by energy exhaustion. An adaptive scheduling strategy is presented to increase the energy efficiency of each sensor node in [61]. However, the larger energy is consumed in the algorithm compared that with the sensor selection procedure. A Distributed smart sensor scheduling is proposed to switch the working mode of sensors automatically in [63]. The authors in [66] proposed an adaptive algorithm by changing the sampling interval when the tracking accuracy is satisfactory, which is focused on tradeoff between communication rate from the temporal domain. A proper criterion about the value of measurements residual for sensors figuring out whether transmitting their measurements is presented in [96]. Base on the criterion, artificial measurements are also created for those unsent measurements to guarantee the tracking accuracy. Furthermore, the best group of task sensor is determined by the generalized Breiman, Friedman, Olshen, and Stone (GBFOS) algorithms. The adaptive algorithm is focused on tradeoff between communication rates from the spatial domain.

To reduce the energy, only part of sensors need to participate into tracking. In general, in 3D underwater environment, four sensors can determine the position of one target. Therefore, some researches are focused on designing the sensor scheduling strategies which can select the most informative sensors from the candidate ones located within the sensing region of the target. In [84], only 60% sensors located closer to the target can receive the waking message. The measuring accuracy of underwater acoustic sensors is dependent on the measuring distance. It is intuitive to select the nearest sensors as the task sensors. However, the algorithm merely based on distance neglect the influence of node topology on the tracking performance. The authors in [97] derive the multiple-model posterior Cramér-Rao lower bound (PCRLB) in the presence of multiplicative noise. Base on the estimated PCRLB, an effective sensor selection scheme is developed for determining the subset sensors to attend tracking task. The algorithm shows great performance on tracking 3D maneuvering target and can be extended to underwater application. Most of the scheduling strategies mentioned before are based on global information. The global sensor scheduling schemes assume that each sensor knows the position of global sensors, which is impractical for underwater acoustic tracking for the limited energy and memory source. The sensor scheduling strategy based on local node selection is proposed in [98]. The communication radius of sensors is assumed adjustable. The fusion center calculates the longest distance between itself and the sensor with probability of detecting the target at next time. The fusion center adjusts the communication radius according to the longest distance and transmits the waking message containing containing the prediction state of the target. These sensors receiving waking messages can further decide whether participating into tracking by calculating its distance to the predicted position.

##### Quantized Methods

Although the raw measurements can provide more accurate information about the target, the limited communication bandwidth and energy make quantized measurements preferable in underwater environment. There is a large amount of papers focus on quantizer design in WSNs [34,69]. On the condition of fixed allowable energy for transmitting a message, the authors in [99] investigate the optimal number of quantization bits and energy allocation strategy to minimize the error of reconstruction. A new distributed quantization method is proposed in [100]. The sensor can automatically adjust its quantization threshold based on the earlier transmission from other sensor nodes. However, the adaptive adjustment of step size, which determines the value of quantization threshold, need further research. Some researchers investigate optimal quantization methods from the aspect of information gain [101,102]. Except for the quantized methods with fixed threshold, the proposed algorithms in WSNs have the common weak in relying on the target current state and requiring heavy computation burden. In underwater target tracking, there are only a few studies on quantized methods. The authors in [67] investigate the influence of node topology on the under tracking performance with quantized measurements. However, the quantized threshold is fixed. Considering most quantization methods in WSNs need complex computation, a simplified objective function for determining the optimal quantization threshold offline is proposed in [103]. By minimizing the expectation of additional error covariance incurred by quantized measurements, the function optimal quantization factor is derived. The factor can be predetermined because it is independent on the sensor location and target state, which significantly eases the computation burden on sensors. The existing WSN quantized methods can be extended to the underwater environment, although constraints such as energy and communication bandwidth are more serious underwater than in WSNs. Optimal quantization methods, especially for underwater acoustic target tracking, need further research. Traditionally, when it comes to the tracking results optimization, filtering techniques are the first choice while other optimization methods have been ignored. However, there is a great attempt to consider technologies from other subjects to improve the tracking performance. Through the simple description of the arithmetic average and the Sage-Husa model, the researchers can find ideas for tracking optimization not constrained to the filtering techniques. Moreover, the relevant sources of application scenarios must also be considered. That’s why we take the tracking optimization method as a classification criterion. Then we compare these papers in Table 5 in the aspect of precision, computation complexity, tracking response time, and corresponding disadvantages. As Table 5 shows, the quantization issue in underwater acoustic tracking have not well studied. However, the bandwidth constraint is much serious in marine environment. More attention should be paid to investigating the quantized methods suitable for underwater tracking

## 6. Discussion, Challenges, Countermeasures, and Lessons Learned

In this section, we present a comparison between all algorithms mentioned before and the challenges in underwater target tracking, including low target detection probability, sound speed variation, eliminating PS ambiguity as well as the balance between tracking accuracy and energy consumption are illustrated. Countermeasures for the mentioned challenges are also presented. The lessons learned about designing the underwater acoustic tracking algorithms also are introduced for readers’ reference.

### 6.1. Discussion

Underwater target tracking plays a crucial part in modern civil and military systems. Therefore, numerous research results have been reported. Due to the special characteristics of the marine environment, the acoustic wave becomes the only appropriate carrier to realize underwater target tracking. In this paper, we make a comprehensive study of the underwater acoustic target tracking algorithms and propose a new taxonomy based on the methods used, namely, instrument-assisted method, mode-based method, and tracking optimization method. The varieties of underwater target tracking algorithms introduced in Section 5 are summarized in Table 6 and Table 7. 

Different from the tables in Section 5, we summarize and compare all these algorithms based on some other aspect: the number of the target, the dimension of the target state, tracking maneuvering or non-maneuvering target. Due to the large amount of papers, we split the summary table into two, based on the tracking mode. As Table 6 and Table 7 show, most tracking algorithms employ active mode, which seems contradictory to what was introduced in Section 2, but actually it is not so, and most researchers prefer using active mode because it is much easier to implement compared that adopting passive mode. However, it is more advocated for researchers to consider the applicability of the algorithm in the actual environment not just for simplicity.

### 6.2. Challenges

Due to the special and complex underwater environment characteristics, the acoustic wave becomes the only suitable carrier for realizing underwater target tracking. However, the problem of acoustic communication and the complexity of the marine environment pose great challenges to target tracking:

*The low target detection probability*: Due to the complexity of the underwater environment, tracking using imaging sensors has the problem of low contrast and visibility which even makes it hard for humans to detect the presence of a target. Tracking using acoustic echoes also has difficulty in detecting due to the attenuation of the acoustic waves.

*Sound speed variation*: Many tracking algorithms based on distance measurements obtained by TDOA or TOA assume that the acoustic waves are transmitted at a constant speed in the marine environment, which is impractical and results in large tracking errors, especially in trilateration. Therefore, how to construct a stable and accurate sound speed model is a challenge.

*PS ambiguity*: When using a sensor array to realize underwater target tracking, PS ambiguity appears due to the structural symmetry of the array. Because the ambiguity makes tracking algorithms more complicated and results in noticeable performance degradation, the process of removing such artifacts is dispensable.

*The balance between tracking accuracy and energy consumption*: If USWNs are exploited for tracking, the energy consumption is a key factor. However, as Section 5 introduces, reducing the energy cost means sacrificing certain tracking accuracy. There are few papers taking both into consideration to design tracking algorithms. It is still a challenge to design a tracking algorithm which can automatically adjust the energy consumption and the tracking accuracy according to the application requirement.

### 6.3. Countermeasures

*Improve low target detection probability*: In most tracking systems, the target is always at a large distance relative to the detecting sensors. With the development of acoustic concealment technology, detecting and tracking targets with low SNR has become an urgent problem. Compared with detect-before-track (DBT), TBD is the feasible technology to solve the problem [96]. Aimed at solving the problem of real-time detecting and tracking multi-target, a non-motorized multi-weak target detection algorithm based on PF-TBD is proposed in [104], which transfers TBD from batch processing to recursive processing. Moreover, the histogram probably multiple hypothesis trackers (H-PMHT) which develops based on traditional TBD has higher real-time [105]. In addition, finite set statistics (FSS) is introduced in [106] to realize number-varying multi-target TBD.

*Sound speed model*: The undefined parameters in water environments such as depth, temperature, and salinity variations have an obvious impact on the sound speed change. The relationship between sound speed variation and these factors is complicated. The authors in [87] adopt one of the relationships described in [107] to construct a sound speed model. The simulation result demonstrates that the tracking accuracy of this algorithm is significantly improved compared with that using constant speed sound. However, this model has limitations of universality. A new calculation of sound speed suitable for all ocean environments is proposed in [108], which is a function of temperature, depth, salinity, and latitude. The authors in [109] have demonstrated the efficiency of the calculation in underwater localization. Hence, the problem of sound speed variation in underwater target tracking can be addressed using the proposed equation in [108].

*Eliminate PS ambiguity*: There are some papers focused on addressing this problem using a multiline array, e.g., twin array and triplet array [110,111]. Nevertheless, the multiline array requires a larger number of hydrophones, which is not acceptable in ASW applications, especially in the scenario using AUVs. The single linear array is preferred in underwater applications for the simplicity of deployment. Firstly, the ambiguity can be eliminated using a single platform by turning the heading angle. For example, if the angle of the target relative to the array becomes larger when the array turns some degrees to the right, the target can be determined on the port side of the array, and vice versa. This approach has the requirement of the target moving at a low speed and in a long-range relative to the tracking platform. Another approach adopts multiple platforms towing line array to remove the ambiguity using the spatial diversity in obtained data. True contacts always locate around the target, while ghost contacts are at the specific position with respect to the heading of the array. By using multiple arrays, the contacts locating at different positions in the region can be classified as ghost contacts. In addition, the authors in [38] propose an optimal disambiguation way by deriving the full Bayesian posterior PDF of the target without bringing any extra cost. In future, the active sensor array can be used to eliminate PS ambiguity by obtaining range information.

*Keep a balance between energy consumption and tracking accuracy*: The authors in [65,66] consider the energy consumption and tracking accuracy simultaneously. In most of tracking algorithms, the calculated position of the target has an inevitable error. Improving the tracking accuracy is more significant in practical applications and practical needs. However, improving the accuracy means fusing more sensor data, which brings large energy consumption. Therefore, we should construct a weighted equation between the energy consumption and the accuracy when designing the tracking algorithms. Through large simulation experiments, the approximate relationship between both factors can be derived. Base on the relationship, the tracking algorithm can automatically adjust the number of sensors transmitting information to meet the requirement of specific application scene.

### 6.4. Lessons Learned

Designing effective underwater target tracking algorithms should take many factors into account especially the effect of the marine environment. Therefore, many additional measures should be employed. Here we list some lessons based.

The medium used in underwater target tracking is acoustic waves; however, the tracking instruments can be varied, as Section 3 introduces. Therefore, how to choose the tracking instrument according to the realistic application is important. In this paper, we classified the tracking algorithms based on the different tracking instruments and compared the strength and weakness of the three classical tracking instruments in Table 3. To sum up, the acoustic imaging sensor is usually used for tracking targets in close range and has the advantage of obtaining not just kinematic information, while having the drawback of limited FOV. TASA and USWNs can track targets whatever in close or distant range while having the problem of PS ambiguity and energy consumption respectively. Researchers can select the appropriate tracking instrument based on their advantages and disadvantages.

Due to the complexity of the marine environment, errors are inevitable in tracking results. Designing a calibration method and choosing proper filter algorithms can reduce the error. The authors in [74] design a pre-processor to reduce the error of obtained bearing information, which makes a great improvement of the tracking performance. In addition, as Table 6 shows, almost every tracking algorithms use some methods for optimization. In Section 5, the tracking algorithms are classified based on the tracking optimization and the corresponding disadvantages are listed in Table 5. Researchers can study these references to find some ideas. Except for improving tracking accuracy, the optimization methods can be designed from the constrained source. 

## 7. Conclusions

With the increasing interest in the exploration of marine resources, underwater target tracking technology has aroused wide attention. Due to the specificity and complexity of the marine environment, acoustic waves have become the most widely used medium for tracking underwater targets. Numerous research papers have reported their tracking results. In this paper, we survey many acoustic tracking algorithms based on a new taxonomy, namely, instrument-assisted methods, mode-based methods, and tracking optimization methods. The contributions of this paper include: (1) It is the first review article about underwater acoustic target tracking; (2) the relevant knowledge is clarified in detail, which makes it easier for the readers to understand the area of underwater target tracking; (3) comprehensive comparisons have been made to make the readers quickly find a suitable method to improve tracking performance; (4) to compensate for the existing shortcomings, review papers are only centered on target tracking based on WSNs.

The future work in this area can focus on several aspects: (1) *Multiple target tracking*. Few of the algorithms previously mentioned in Table 6 and Table 7 can be used to track multiple targets. However, multiple target tracking methods are important in real application. For example, according to the developing trend of modern naval warfare, ships and submarines in the future will be in the form of unit operations. Hence, objects like torpedoes will present in multiple forms. The multiple underwater target tracking technology is the future research area for the scientific and technological personnel. (2) *Enhancing measurement accuracy of passive tracking*. In ASW applications, the target of interest is usually far from the tracker; the tracker can only passively listen to the signal radiated from the target for concealment. Furthermore, the development of silent technologies makes it hard to receive signals from target for passive tracking. Even if the signal is detected successfully, if the passive tracker has a low accuracy when measuring bearing information about the target, the tracking performance will degenerate significantly. (3) *Data fusion*. The tracking system will tend to the multi-state system. Therefore, how to make an effective fusion of homogeneous or heterogeneous measurements information to achieve more robust and accurate tracking will be a research hot issue. (4) *Motion strategies for mobile sensors*. Underwater target tracking is usually realized in large scale areas. Sensors with mobility outperform static sensors. Considering the influence of topology, how to design effective coordinate motion strategies for sensor is a hot issue. On this basis, the adaptive motion strategies based on local information can be developed. (5) *Quantized methods*. As mentioned in Section 5.3, there are few underwater acoustic target tracking algorithms that consider the quantization issue. However, the limited bandwidth in underwater channel makes it impossible to transmit raw measurements.

## Figures and Tables

**Table 1:** Comparison of survey papers for target tracking based on WSNs.

Reference	Survey Content	Reference Number	Published Year	The Related Knowledge
[11]	The whole process of target tracking	High	2016	Detailed (The whole process of tracking, the metrics for analyzing algorithms, the requirement of tracking based on WSNs)
[12]	The management of energy for target tracking	Medium	2012	Brief
[13]	The prediction algorithms used in target tracking	Low	2014	Brief
[14]	The target recovery techniques in target tracking	Low	2016	Brief
[15]	The security of target tracking	Low	2014	Brief

**Table 2:** Comparison of survey papers for target tracking.

Reference	Taxonomy	Reference Number	The Newest Reference	Standard of Comparison
Ours	Instrument-assisted method	High	2017	Comprehensive (tracking precision, cost, complexity, note, the characteristic of target)
	Mode-based method			
	Tracking optimization method			
[11]	Network structure	High	2015	Common (prediction, energy management, target recovery)
	Problem formulation			
	Number of targets			
	Type of target			
[18]	Network structure	Low	2015	Only summary no comparison
	Prediction-based			
	Type of objects			
	Type of sensors			
	Number of targets			
	Recovery			

**Table 3:** Summary of tracking instrument-assisted methods.

Tracking Instrument	Reference	Precision	Complexity	Cost	Strength	Weakness
Acoustic imaging sensor	[49]	Medium	Medium	Medium	Have considerable tracking performance in close range. Obtain not only motion information but also features	Low contrast Low visibility Low LFR
	[50]	High	Low	Low		
	[52]	Medium	Low	Low		
	[53]	High	Medium	Medium		
TASA	[38]	High	Low	Low	Obtain accurate bearing information about the target	Port-starboard ambiguity Tracking performance depends on the relative position between the target and the array
	[55]	High	Medium	Low		
	[56]	High	Low	High		
	[58]	Low	Medium	Low		
UWSNs	[61]	Medium	Low	Low	Wide-range distributed Real-time Self-organization Low cost Rapid deployment Fault-tolerance	Limited energy Limited communication ability Tracking security
	[62]	High	Medium	Low		
	[63]	Medium	Medium	Low		
	[65]	High	Medium	Low		
	[66]	Medium	Medium	Low		
	[67]	High	Medium	Low		
	[68]	Medium	Medium	Low		
	[71]	—	High	Low		
	[72]	—	Medium	Low		

**Table 4:** Summary of tracking mode-based methods.

Mode	Reference	Precision	Tracking Technology	Measurements Received
Passive	[37]	High	ADCSP, data fusion, PF	TDOA
	[56]	Medium	MEGEKF/UKF, data fusion	DOA
	[73]	Medium	IUKF, consensus estimation	DOA
	[74]	High	Pre-processing of noise, UKF, IUKF	DOA
	[75]	High	EKF	DOA, TDOA
	[77]	High	KF, AR, SD-CFAR	DOA
	[78]	High	EM, EKF, UKF	DOA
	[80]	Medium	EKF	TDOA
Active	[38]	Medium	Control strategy for AUV A branch and bound technique	DOA, TOA
	[81]	High	FCM, KF	RSSI
	[82]	Medium	3DUT, BND	TDOA
	[83]	High	CEUTT	TDOA

**Table 5:** Summary of tracking optimization methods.

Method	Reference	Precision	Complexity	Response	Disadvantages
KF	[60]	Medium	Medium	Medium	2-D targets
	[70]	Medium	High	Quick	Increase the computation burden
	[74]	High	Low	Quick	The linearity is limited to tracking target in long-distance
	[75]	High	Medium	Quick	Sensitive to the accuracy of the estimation of the initial target.
	[81]	High	Low	Quick	Extra energy consumption
	[84]	Medium	Medium	Medium	Being suitable for deep underwater scene
	[85]	Medium	Medium	Quick	Ignore the energy consumption of UWSNs Have poor performance for maneuvering targets
PF	[86]	Medium	Medium	Medium	Poor performance for maneuvering target
	[87]	High	Medium	Medium	The performance for experimental data is not as good as simulation results
	[90]	Medium	Medium	Medium	Only has simulation results
	[91]	High	Medium	Quick	Choosing KF as the comparison is not proper
Arithmetic average	[70]	Medium	Low	Quick	The proper order of pre-processor is hard to decide
Sage-Husa model	[71]	High	Medium	Quick	Being sensitive to the measurement error
Sensor scheduling strategies	[61]	Medium	Low	Quick	Waking up all senors with the probability of detecting the target
	[63]	Medium	Medium	Quick	Only validated for non-maneuvering target
	[66]	Medium	Medium	Medium	The sampling interval set is small
	[84]	Medium	Low	Quick	The ratio of waking sensor is determined without theory support
	[96]	High	High	Medium	The fusion center is ambiguous.
	[98]	Medium	Low	Medium	Assuming the communication radius is adjustable
Quantized methods	[67]	Medium	Low	Medium	The quantized threshold is fixed
	[103]	High	Medium	Medium	The fusion center is ambiguous

**Table 6:** Summary and comparison of underwater passive acoustic target tracking algorithms.

Reference	Instrument-Assisted	Optimization	Target		
[37]	UWSNs	EKF	Single	3-D	No
[56]	TASA	EKF	Single	2-D	Yes
[58]	TASA	—	Multiple	3-D	No
[63]	UWSNs	KF	Single	2-D	No
[67]	UWSNs	PF, Quantization	Single	2-D	No
[73]	UWSNs	UKF	Single	2-D	Yes
[74]	TASA	Arithmetic average	Single	2-D	No
[75]	TASA	Sage-Husa model	Single	2-D	No
[77]	TASA	KF	Multiple	2-D	No
[78]	—	KF	Single	2-D	Yes
[80]	—	EKF	Single	3-D	Yes

**Table 7:** Summary and comparison of underwater active acoustic target tracking algorithms.

Reference	Instrument-Assisted	Optimization	Target		
[38]	TASA	—	Single	2-D	Yes
[49]	Acoustic imaging sensors	—	Multiple	3-D	Yes
[50]	Acoustic imaging sensors	EKF	Multiple	3-D	Yes
[52]	Acoustic imaging sensors	KF	Single	3-D	No
[53]	Acoustic imaging sensors	—	Multiple	3-D	No
[55]	TASA	PF	Single	2-D	No
[61]	UWSNs	KF	Single	2-D	No
[62]	UWSNs	KF	Single	2-D	Yes
[65]	UWSNs	KF	Single	2-D	No
[66]	UWSNs	EKF	Single	3-D	Yes
[68]	UWSNs	—	Single	3-D	No
[81]	—	KF	Single	3-D	Yes
[82]	UWSNs	—	Single	3-D	Yes
[83]	UWSNs	—	Single	3-D	Yes
[84]	UWSNs	KF	Single	3-D	Yes
[87]	—	EKF	Single	3-D	No
[88]	—	EKF	Single	2-D	Yes
[94]	Acoustic imaging sensor	PF	Single	3-D	Yes
[103]	UWSNs	PF, Quantization	Single	3-D	Yes
